# Carboxyl-Terminal Residues N478 and V479 Required for the Cytolytic Activity of Listeriolysin O Play a Critical Role in *Listeria monocytogenes* Pathogenicity

**DOI:** 10.3389/fimmu.2017.01439

**Published:** 2017-11-01

**Authors:** Changyong Cheng, Li Jiang, Tiantian Ma, Hang Wang, Xiao Han, Jing Sun, Yongchun Yang, Zhongwei Chen, Huifei Yu, Yi Hang, Fengdan Liu, Bosen Wang, Weihuan Fang, Huarong Huang, Chun Fang, Chang Cai, Nancy Freitag, Houhui Song

**Affiliations:** ^1^College of Animal Science and Technology of Zhejiang A&F University, China-Australian Joint Laboratory for Animal Health Big Data Analytics, Zhejiang Provincial Engineering Laboratory for Animal Health Inspection & Internet Technology, Lin’an, China; ^2^Zhejiang University Institute of Preventive Veterinary Medicine, Zhejiang Provincial Key Laboratory of Preventive Veterinary Medicine, Hangzhou, China; ^3^College of Biological and Environmental Science, Institute of Developmental and Regenerative Biology, Hangzhou Normal University, Hangzhou, China; ^4^College of Animal Science, Yangtze University, Hubei, China; ^5^School of Veterinary and Life Sciences, Murdoch University, Murdoch, WA, Australia; ^6^Department of Microbiology and Immunology, University of Illinois at Chicago College of Medicine, Chicago, IL, United States

**Keywords:** *Listeria monocytogenes*, listeriolysin O, cytolytic activity, virulence, live vaccine

## Abstract

*Listeria monocytogenes* is a facultative intracellular pathogen that secretes the cytolysin listeriolysin O (LLO), which enables the bacteria to cross the phagosomal membrane. *L. monocytogenes* regulates LLO activity in the phagosome and minimizes its activity in the host cytosol. Mutants that fail to compartmentalize LLO activity are cytotoxic and have attenuated virulence. Here, we showed that residues N478 and V479 of LLO are required for LLO hemolytic activity and bacterial virulence. A single N478A mutation (LLO_N478A_) significantly increased the hemolytic activity of LLO at a neutral pH, while no difference was observed at the optimum acidic pH, compared with wild-type LLO. Conversely, the mutant LLO_V479A_ exhibited lower hemolytic activity at the acidic pH, but not at the neutral pH. The double mutant LLO_N478AV479A_ showed a greater decrease in hemolytic activity at both the acidic and neutral pHs. Interestingly, strains producing LLO_N478A_ or LLO_V479A_ lysed erythrocytes similarly to the wild-type strain. Surprisingly, bacteria-secreting LLO_N478AV479A_ had barely detectable hemolytic activity, but exhibited host cell cytotoxicity, escaped from the phagosome, grew intracellularly, and spread cell-to-cell with the same efficiency as the wild-type strain, but were highly attenuated in virulence in mice. These data demonstrate that these two residues are required for LLO hemolytic activity and pathogenicity in mice, but not for escape from the phagosome and cell-to-cell spreading. The finding that the nearly non-hemolytic LLO_N478AV479A_ mutant grew intracellularly indicates that mutagenesis of a virulence determinant is a novel approach for the development of live vaccine strains.

## Introduction

The Gram-positive, facultative intracellular bacterium *Listeria monocytogenes* is the causative agent of listeriosis, a severe foodborne infection with a high mortality rate (1). This pathogen is ubiquitous, and it has been isolated from humans and animals, as well as from raw and ready-to-eat foods (2, 3), and it is capable of invading a wide variety of eukaryotic cells, including endothelial cells and macrophages (4, 5). Each step of a successful infection established by *L. monocytogenes* is highly dependent upon the production of virulence-associated factors (1, 6, 7). Among these virulence factors, listeriolysin O (LLO, encoded by the *hly* gene) plays a central role in the escape of bacteria from the phagosomal compartment, and it is also involved in cell-to cell spreading (8), thus making LLO an essential determinant of *L. monocytogenes* pathogenesis. LLO belongs to the family of cholesterol-dependent cytolysins (CDCs) that are secreted by many pathogenic Gram-positive bacteria (9), and CDCs are the largest family among the bacterial pore-forming toxins (10, 11). Other members include more than 20 pore-forming toxins produced by different bacterial species, like anthrolysin O, streptolysin O, perfringolysin O (PFO), and pneumolysin. All CDCs are secreted as soluble monomers by their cognate bacteria and are characterized by their ability to bind to the cholesterol of host membranes and form large pores (8).

The most heterogeneous and better studied region of CDCs is the amino (N)-terminal sequence, which harbors distinct functions for some of the family members (11). *L. monocytogenes* LLO is composed of 529 residues and possesses at its N-terminus a 25-residue-long typical signal peptide. To maintain its intracellular niche, *L. monocytogenes* must restrict the pore-forming activity of LLO to the phagosomal compartment and prevent perforation of the plasma membrane. Correct compartmentalization of LLO activity requires a 26-amino-acid sequence located in the extreme N-terminus of the protein, which resembles a eukaryotic PEST-like sequence that is rich in the amino acids proline, glutamate, serine, and threonine (12, 13). Mutants lacking the PEST-like region exhibit higher intracellular LLO levels and increased cytotoxicity, and, therefore, result in a higher permeability of the host plasma membrane, which consequently decreases the virulence of the bacteria because they are no longer able to evade host extracellular defenses (4, 13, 14). Recently, Koster and colleagues determined the crystal structure of natively produced *L. monocytogenes* LLO, which showed that the N-terminal PEST-like sequence forms a left-handed polyproline type II helix that is involved in intra- and intermolecular interactions (11). Among the pore-forming toxin family members, LLO is the only cytolysin that is made by an intracellular pathogen, and it has a pronounced acidic pH optimum (pH 5.5) (15), which is attributed to the sophisticated regulation of its activity *via* a pH sensor consisting of the three acidic residues E247, D208, and D320 (the “acidic triad”) (16). However, PFO does not have a pronounced acidic pH optimum, but rather is active at both acidic and neutral pHs. Moreover, the high cytotoxicity of secreted PFO that strongly permeabilizes the host cell is caused in part by the lack of a PEST-like sequence, which targets LLO for phosphorylation and degradation in the cytosol (12). As a result, *L. monocytogenes* expressing PFO in place of LLO is capable of escaping from a host vacuole *in vitro*, but is unable to grow intracellularly and is non-virulent (17). Thus, LLO is unique, not in its ability to mediate vacuolar escape, but in its lack of host-cell toxicity. Interestingly, the level of LLO hemolytic activity does not correlate with the efficiency of *Listeria* escape from the vacuole within host cells. A previous study employed an intracellular genetic selection to isolate mutants in PFO that supported the intracellular growth of *L. monocytogenes*, and it identified several PFO mutants containing a single amino acid change that had low or undetectable hemolytic activity. Nevertheless, these non-hemolytic mutants were still capable of escaping from the phagocytic vacuole of J774 macrophages, albeit less efficiently than the wild-type strain (18).

In comparison to the thoroughly studied N-terminus of CDCs, little is known about the carboxyl (C)-terminus of this protein family. So far as we know, all CDCs contain a highly conserved undecapeptide (ECTGLAWEWWR) at their C-terminus, which was originally thought to be critical for cholesterol-mediated membrane recognition, as mutations in it abolished pore formation (19). However, the mechanistic contribution of this domain is unclear. Recently, it was demonstrated that the undecapeptide was not responsible for cholesterol binding. Instead, a threonine–leucine pair in the C-terminal part of the protein was important (20). In fact, the conserved undecapeptide was shown to be a key structural element that allows the correct conformation of the cholesterol-binding motif (21). In the present study, we used site-directed mutagenesis to identify a double-residue mutant (LLO_N478AV479A_) close to the undecapeptide region of LLO that rendered LLO almost inactive, yet, still mediated vacuolar escape, intracellular growth, and cell-to-cell spreading with an efficiency close to that of the wild-type strain, even though it was completely non-virulent in mice. These results demonstrate that these two residues are active sites that are required for LLO hemolytic activity and, therefore, are essential for *Listeria* infections of mice. Here, we addressed the role of this region in the biological activities of LLO and concluded that the virulence, but not the intracellular fate, of *L. monocytogenes* directly correlated with LLO hemolytic activity. Furthermore, the attenuated virulence of the LLO_N478AV479A_ mutant suggests that it has great potential as a live vaccine vehicle.

## Results

### Residues N478 and V479 within LLO Are Required for Hemolytic Activity

**Figure 1:**
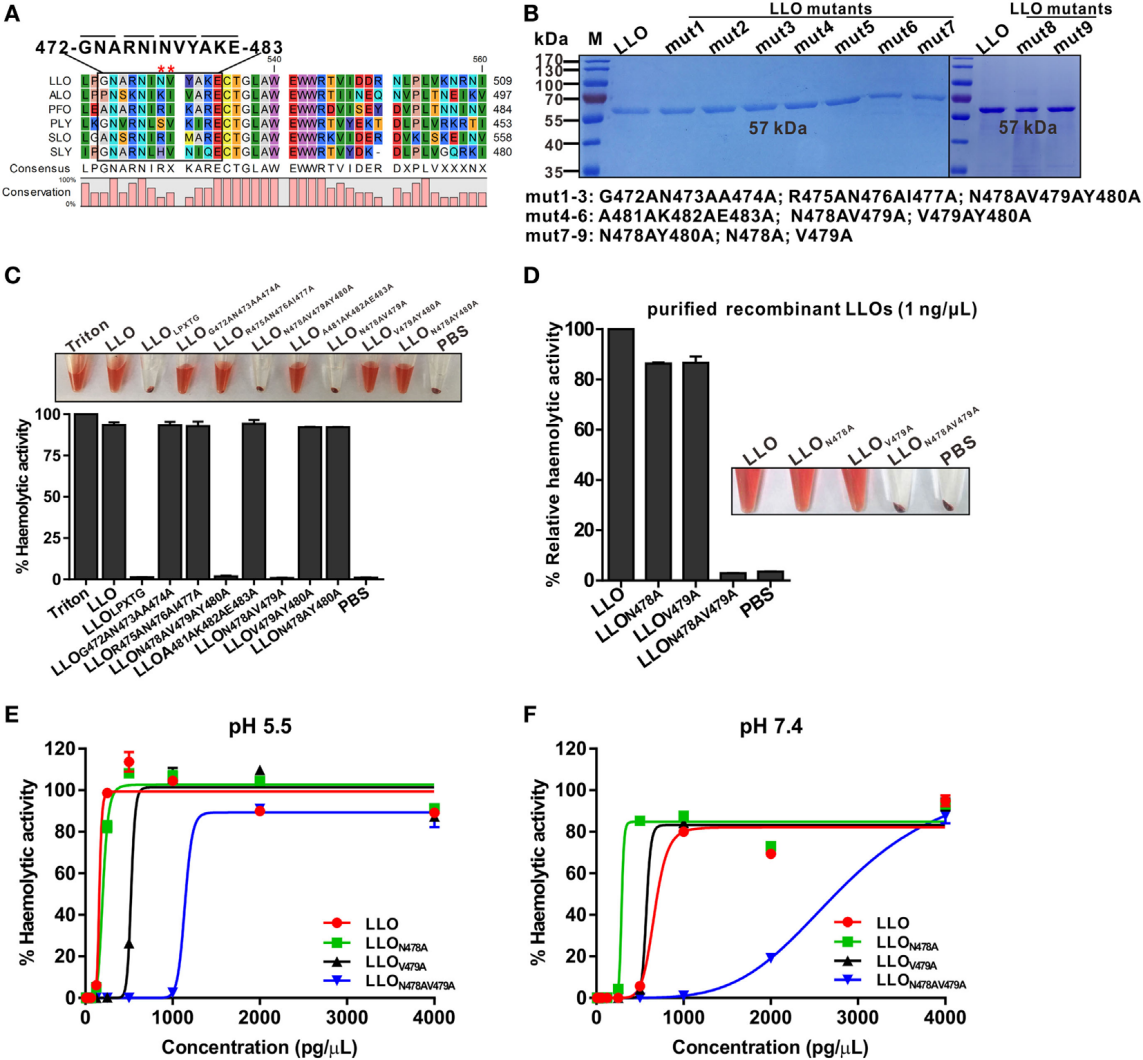
Residues N478 and V479 of listeriolysin O (LLO) are required for hemolytic activity. **(A)** The strategy for the generation of a mutated LLO with a 12-amino-acid in-frame deletion (472–GNARNINVYAKE–483) that could form an LPXTG motif. Stars indicate the identified key residues, N478 and V479, of LLO. The listed toxins are from the following organisms: LLO, *L. monocytogenes* listeriolysin; ALO, *Bacillus anthracis* anthrolysin; PFO, *Clostridium perfringens* perfringolysin; PLY, *Streptococcus pneumoniae* pneumolysin; SLO, *Streptococcus pyogenes* streptolysin; SLY, *Streptococcus suis* suilysin. **(B)** Sodium dodecyl sulfate–polyacrylamide gel electrophoresis analysis of the purified histidine-tagged recombinant LLO and its mutated forms expressed in *Escherichia coli*. **(C,D)** Comparison of the hemolytic activity of the LLO mutants relative to wild-type LLO. Erythrocytes incubated with 1% Triton X-100 or phosphate-buffered saline (PBS) served to determine the maximum (100%) and minimum (0%) hemolytic activity, respectively. **(E,F)** Hemolytic activity of the identified LLO mutants, LLO_N478A_, LLO_V479A_, and LLO_N478AV479A_, at various concentrations (0–4 ng/µL) at pH 5.5 **(E)** and 7.4 **(F)**. Erythrocytes incubated with 1% Triton X-100 or PBS served to determine the maximum (100%) and minimum (0%) hemolytic activity, respectively. Data in C, D, E, and F are expressed as means ± SDs of three independent experiments.

We previously generated an *L. monocytogenes* mutant strain for intracellular antigen-presenting containing a 12-amino-acid in-frame deletion (472–GNARNINVYAKE–483) at the C-terminus of LLO that could form an LPXTG motif (Figure 1A), a characteristic C-terminal sorting signal known to direct covalent anchoring to the peptidoglycan of Gram-positive bacteria. Surprisingly, its ability to lyse erythrocytes was completely impaired, suggesting that the deleted 12 residues might be required for LLO cytolytic activity, which prompted us to further investigate which single amino acid among these 12 residues plays a decisive role in modulating the LLO activity. Using site-directed mutagenesis, we generated a series of different combinations of double and triple mutant LLO proteins, which were then expressed as C-terminally histidine-tagged recombinant proteins in *Escherichia coli* and purified to homogeneity by nickel-affinity chromatography to analyze their hemolytic activity (Figure 1B). As expected, we found that among all the LLO mutants, only the mutant proteins LLO_N478AV479AY480A_ and LLO_N478AV479A_ completely lost their hemolytic activity, while the others lysed erythrocytes as efficiently as wild-type LLO (Figure 1C), suggesting that residues N478 and V479 are critical for LLO activity. Based on this observation, we generated two single-amino-acid mutants (LLO_N478A_ and LLO_V479A_) to determine, which residue was essential for controlling the cytolytic activity of LLO. Strikingly, the mutation of either N478A or V479A had little effect on the hemolytic activity of LLO at a concentration of 1 ng/µL, which was sufficient for wild-type LLO to completely perforate erythrocyte membranes and led to full hemolytic activity (Figure 1D). Previous studies established that pore formation by LLO is pH sensitive and concentration dependent at the host temperature (37°C), with LLO being more active at an acidic pH (16). Therefore, the N478A, V479A, and N478AV479A mutants at various concentrations were further tested for hemolysis at pH 5.5 and 7.4. As shown, the LLO_N478A_ mutant had slightly higher LLO hemolytic activity at a neutral pH, while no difference in activity was observed at the acidic pH optimum of this cytolysin, compared with wild-type LLO (Figures 1E,F). Conversely, the mutant LLO_V479A_ exhibited lower hemolytic activity at the acidic pH, but not at the neutral pH. In addition, the LLO_N478AV479A_ mutant showed a 100% decrease in hemolytic activity compared with wild-type LLO at both the acidic and neutral pHs at a low concentration (less than or equal to 1 ng/µL) (Figures 1E,F). However, to our surprise, the native hemolytic activity of LLO_N478AV479A_ was fully restored by increasing its concentration to 2 and 4 ng/µL at pH 5.5 and 7.4, respectively (Figures 1E,F). LLO_N478AV479A_ can be considered as a non-hemolytic LLO mutant mainly for two reasons. One is the fact that wild-type LLO exhibits very efficient hemolytic activity *in vitro* and results in complete erythrocyte lysis at pH 5.5 at a very low concentration of approximately 0.1 ng/µL (~0.25 ng/μL in our study) (11). Another reason is that a similar mutation (LLO_E262K_) rendered LLO virtually inactive at low concentrations, but relatively active at a higher concentration of 1 ng/µL (11). Moreover, LLO and its active mutants have a pronounced acidic pH optimum and remain relatively inactive at neutral pH. Collectively, these results showed clearly that residues N478 and V479 are required for LLO hemolytic activity.

### *L. monocytogenes* Expressing LLO_N478AV479A_ Lacks Hemolytic Activity but Is Cytotoxic to Host Cell Membranes

**Figure 2:**
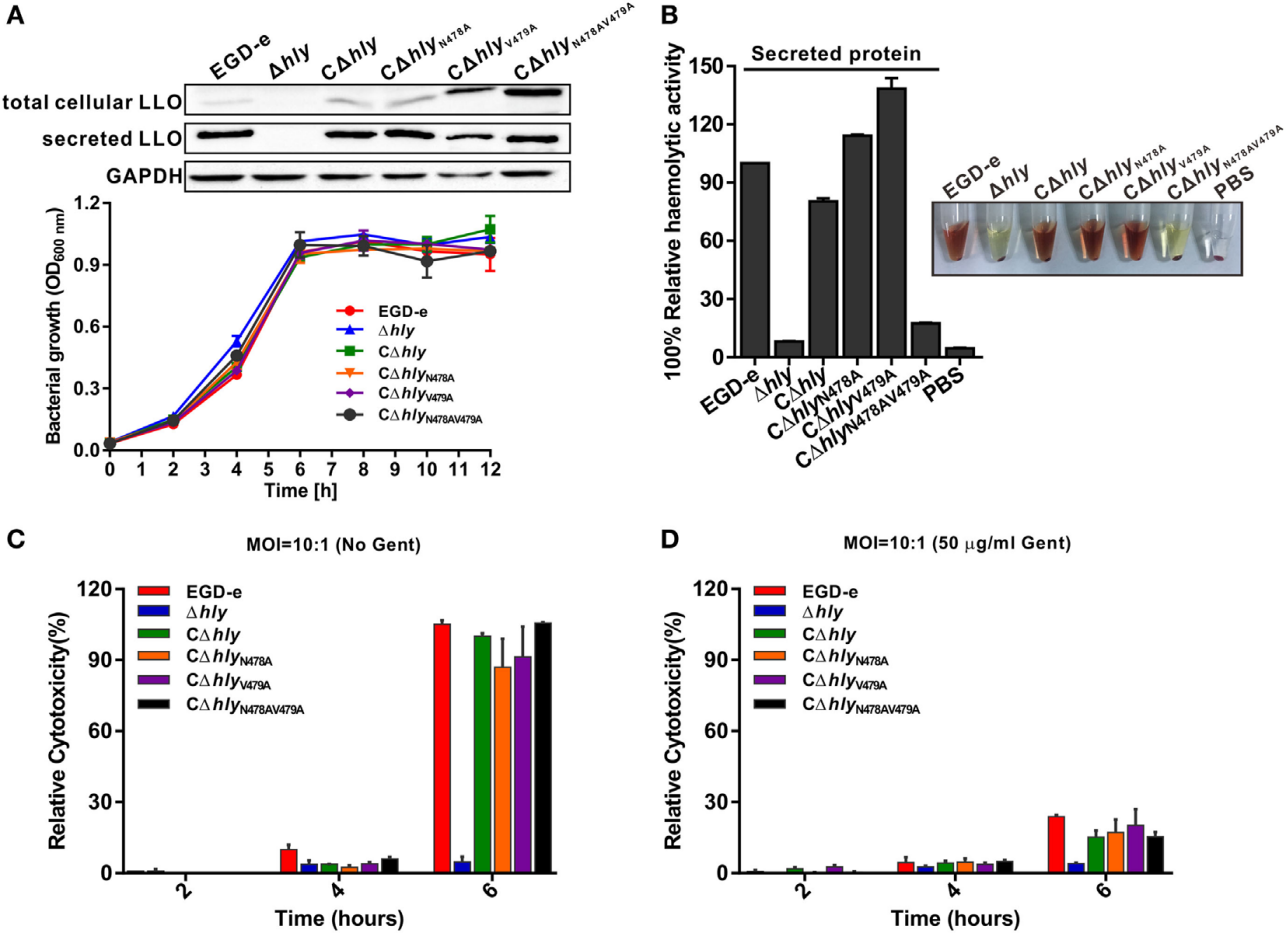
*Listeria monocytogenes* expressing LLO_N478AV479A_ lacks hemolytic activity but is cytotoxic to host cell membranes. **(A)** Secreted listeriolysin O (LLO) was detected by western blotting, and *in vitro* bacterial growth of the *L. monocytogenes* wild-type EGD-e and Δ*hly* strains, and the complemented strains CΔ*hly*, CΔ*hly*_N478A_, CΔ*hly*_V479A_, and CΔ*hly*_N478AV479A_. **(B)** Hemolytic activity of secreted LLO from the culture supernatants of the *L. monocytogenes* wild-type EGD-e and Δ*hly* strains, and the complemented strains CΔ*hly*, CΔ*hly*_N478A_, CΔ*hly*_V479A_, and CΔ*hly*_N478AV479A_. **(C,D)** Lactate dehydrogenase (LDH) release into the tissue culture medium was used to monitor the perforation of the host cell plasma membrane. The percentage of the maximal LDH release from monolayers of J774 macrophages infected with the indicated *L. monocytogenes* strains at 2, 4, and 6 h postinfection in the presence or absence of gentamicin (50 µg/mL) is indicated. All the data are expressed as means ± SDs of three independent experiments.

Having established that the LLO_N478AV479A_ mutation significantly impaired the activity of purified LLO, we further complemented the Δ*hly* mutant strain with wild-type LLO, LLO_N478A_, LLO_V479A_, or LLO_N478AV479A_ under the natural *hly* promoter using the *Listeria* integrative plasmid, pIMK2. As shown by western blotting (Figure 2A), the four resulting mutant strains, CΔ*hly*, CΔ*hly*_N478A_, CΔ*hly*_V479A_, and CΔ*hly*_N478AV479A_, were capable of expressing and secreting LLO or its mutant forms in comparable amounts to wild-type LLO in the culture supernatant, suggesting that these amino acid substitutions did not affect LLO synthesis and secretion. Moreover, we found that these mutations also had no effect on bacterial growth *in vitro* (Figure 2A). Furthermore, the hemolytic activities recorded in the supernatants of the mutants CΔ*hly*, CΔ*hly*_N478A_, and CΔ*hly*_V479A_ were comparable to that of the wild-type EGD-e strain, whereas the CΔ*hly*_N478AV479A_ mutant did not exhibit any detectable hemolytic activity, similar to the Δ*hly* mutant (Figure 2B). Consistent with the hemolytic data obtained from the recombinant LLO variants, we conclude that *L. monocytogenes* expressing LLO_N478AV479A_ is indeed non-hemolytic. To monitor directly the cytotoxicity of the different LLO constructs, we detected the release of a host cytosolic enzyme, lactate dehydrogenase (LDH), into the tissue culture medium from infected J774 macrophages. At early timepoints during the infection (2 and 4 h) with any of these strains, very little detectable LDH was released either in the presence or absence of gentamicin (Figures 2C,D). After 6 h of infection, the amount of released LDH from all the infected cells, except for the avirulent Δ*hly* strain, increased significantly, especially in the absence of gentamicin (Figure 2C). However, to our surprise, the amount of LDH released from macrophages infected with the non-hemolytic CΔ*hly*_N478AV479A_ strain after 6 h of infection was comparable to those of the hemolytic strains, including the wild-type EGD-e, CΔ*hly*, CΔ*hly*_N478A_, and CΔ*hly*_V479A_ strains (Figure 2C), indicating that *L. monocytogenes* expressing LLO_N478AV479A_ lacks hemolytic activity but exhibits normal cytotoxicity to host cell membranes. As expected, when J774 cells were incubated in the constant presence of 50 µg/mL gentamicin, much less LDH was released during a 6-h infection by any strain compared with those in the absence of gentamicin, which was presumably due to the fact that permeabilization of the cells allowed the influx of gentamicin, which then killed the intracellular bacteria (Figure 2D) and prevented further permeabilization and LDH release.

### The N478V479 Mutation of LLO Reduces Virulence in Mice

**Figure 3:**
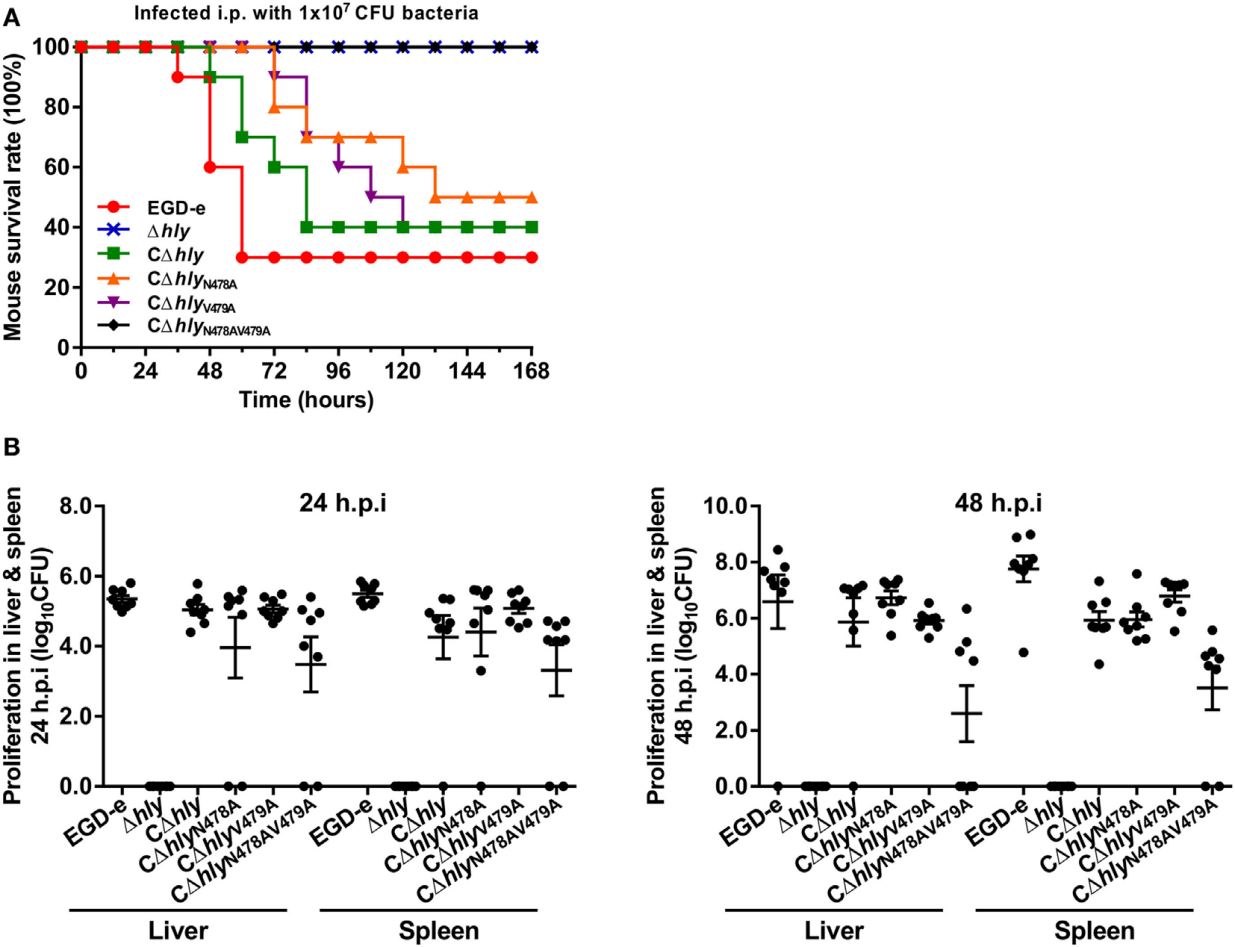
The N478V479 mutation of listeriolysin O reduces virulence in mice. **(A)** The Kaplan–Meier curve represents the survival of ICR mice over time. Ten mice in each experimental group were infected intraperitoneally with 1 × 10^7^ CFU of *Listeria monocytogenes* and monitored for up to 7 days after infection. Data are represented as the percentage survival over time, and significance was determined *via* a log-rank test. **(B)** The *L. monocytogenes* wild-type EGD-e Δ*hly* strains, and the complemented strains CΔ*hly*, CΔ*hly*_N478A_, CΔ*hly*_V479A_, and CΔ*hly*_N478AV479A_ were inoculated intraperitoneally into ICR mice at 5 × 10^6^ CFU. Animals were euthanized 24 and 48 h after infection and organs (liver and spleen) were recovered and homogenized, and the homogenates were serially diluted and plated on brain–heart infusion (BHI) agar. The numbers of bacteria colonizing the liver and spleen are expressed as means ± SDs of the log_10_ CFU per organ for each group.

The virulence of the mutant strains was evaluated in a murine listeriosis model. ICR mice were inoculated intraperitoneally with ~10^6^ bacteria, and their survival was monitored for up to 7 days after infection. To our surprise, all the mice infected with the bacteria synthesizing LLO_N478AV479A_ survived, similar to those infected with the avirulent Δ*hly* strain (Figure 3A). In contrast, infection with the same number of the complemented strain synthesizing wild-type LLO led to 60% mortality (*P* < 0.001), which was comparable to that of the parental strain that exhibited 70% mortality (Figure 3A). Interestingly, the other two complemented strains expressing LLO_N478A_ and LLO_V479A_ resulted in 40 and 60% mortality, respectively, albeit with a relatively low lethal efficiency (Figure 3A). Moreover, the number of colony-forming units (CFU) recovered from the spleens and livers of infected mice after 24 and 48 h of infection was significantly lower (~2–3 orders of magnitude) for the CΔ*hly*_N478AV479A_ mutant compared with the strain expressing wild-type LLO (Figure 3B), indicating that the CΔ*hly*_N478AV479A_ mutant was severely attenuated for virulence, and that mice infected with this mutant exhibited significantly lower bacterial burdens compared with the mice infected with the wild-type strain. As expected, no detectable bacteria were recovered from the organs of mice infected with the avirulent Δ*hly* strain, and the virulence of this mutant was fully restored by complementing it with wild-type LLO, LLO_N478A_, or LLO_V479A_. Taken together, these results establish a critical role for these two residues of LLO in the virulence of *L. monocytogenes*.

### The LLO N478V479 Mutation Does Not Affect the Efficiency of Intracellular Growth in Macrophages

**Figure 4:**
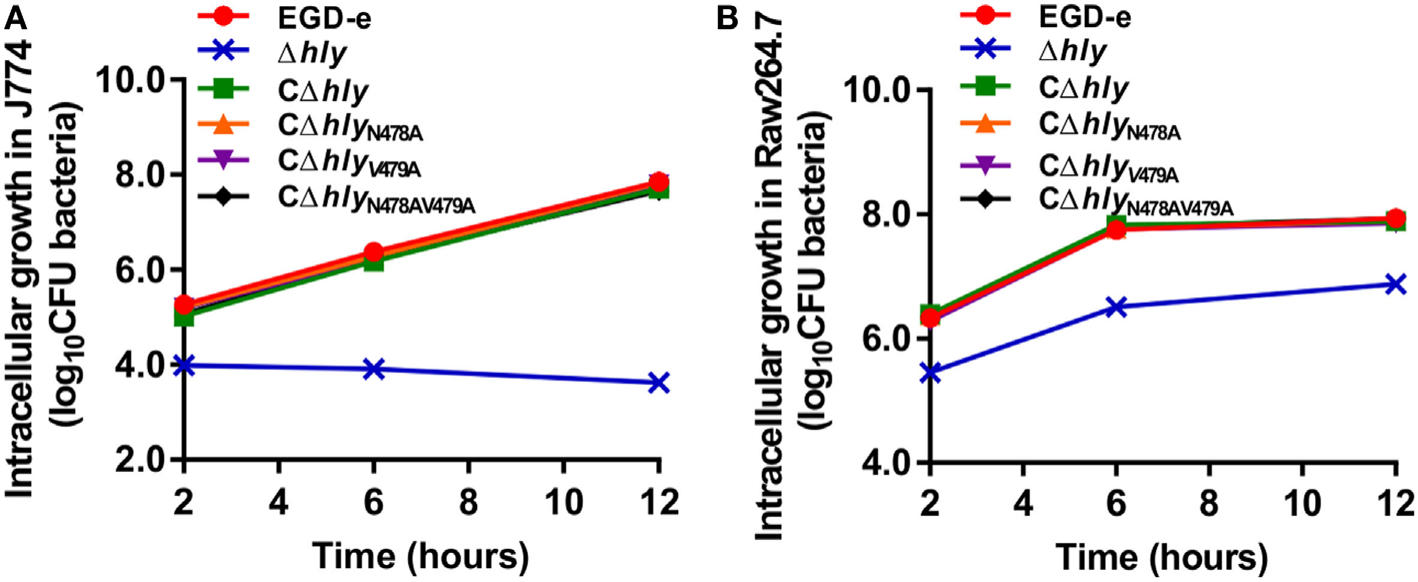
Intracellular growth of *Listeria monocytogenes* strains producing the indicated listeriolysin O proteins in murine-derived J774 and RAW264.7 macrophages. Gentamicin (50 µg/mL) was added 0.5 h postinfection. The J774 **(A)** and RAW264.7 **(B)** cells infected with the *L. monocytogenes* wild-type EGD-e and Δ*hly* strains, and the complemented strains CΔ*hly*, CΔ*hly*_N478A_, CΔ*hly*_V479A_, and CΔ*hly*_N478AV479A_ were lysed at the indicated time points (2, 6, and 12 h), and viable bacteria were serially plated on brain–heart infusion agar plates. The number of recovered bacteria able to invade cells and survive are expressed as means ± SDs for each strain.

We investigated the ability of wild-type *L. monocytogenes* and the LLO mutants to grow intracellularly in murine-derived macrophages J774 and RAW264.7 cells. In this assay, adding the antibiotic gentamicin to the culture medium kills extracellular bacteria, but has no measurable effect on the growth of intracellular bacteria. During the infection process, intracellular proliferation of the LLO-deleted avirulent Δ*hly* strain was almost completely impaired compared with the wild-type strain (Figure 4A). Such compromised bacterial cell proliferation of the Δ*hly* strain was finely restored in the complemented strains CΔ*hly*, CΔ*hly*_N478A_, or CΔ*hly*_V479A_, which secrete the same amount of active LLO as the wild-type EGD-e strain (Figure 4A). Unexpectedly, the Δ*hly* bacteria complemented with an inactive LLO (CΔ*hly*_N478AV479A_) also grew well within J774 macrophages, with an efficiency that was nearly identical to that of the wild-type, CΔ*hly*, CΔ*hly*_N478A_, and CΔ*hly*_V479A_ strains (Figure 4A). To further confirm these data, we conducted this experiment in another murine-derived macrophage cell line, RAW264.7, and the results were completely consistent with those obtained in J774 macrophages (Figure 4B). Therefore, the fact that the mutant strain CΔ*hly*_N478AV479A_, which had barely detectable hemolytic activity, grew intracellularly with the same efficiency as strains with wild-type levels of activity suggests that the LLO N478V479 mutation does not affect the efficiency of intracellular growth in macrophages, and, more interestingly, that the level of hemolytic activity does not correlate with the efficiency of proliferation within macrophages.

### *L. monocytogenes* Expressing LLO_N487V479_ Is Not Defective in Cell-to-Cell Spreading

**Figure 5:**
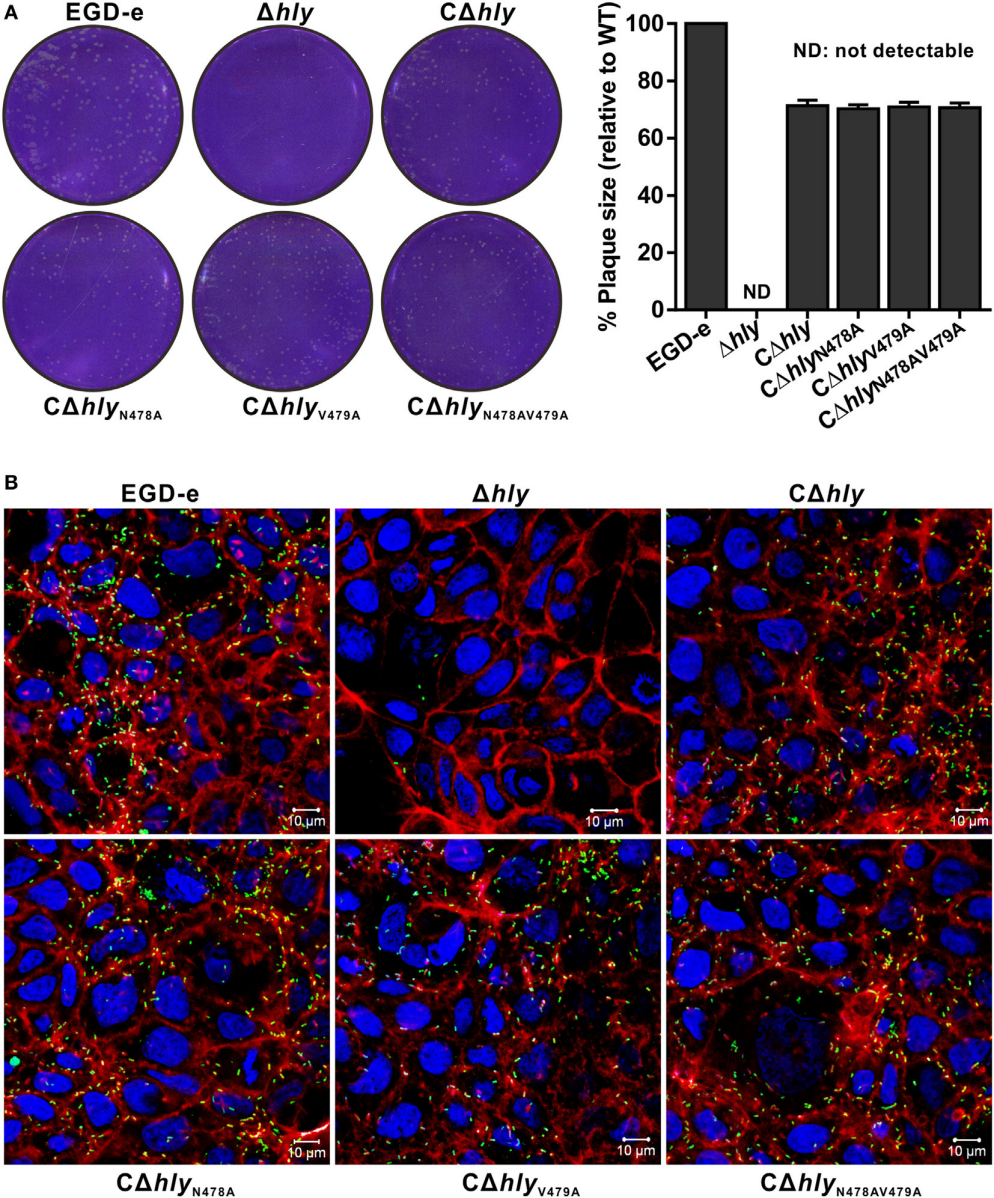
*Listeria monocytogenes* expressing LLO_N487V479_ is not defective in cell-to-cell spreading. **(A)** Plaque sizes formed by the indicated *L. monocytogenes* mutants in L929 cell monolayers as a percentage of the plaque size formed by wild-type bacteria. Following bacterial internalization, gentamicin was added to a concentration of 10 µg/mL as indicated. The values shown represent the means of three independent experiments, and the error bars indicate the SDs. **(B)** Actin tail formation in Caco-2 cells infected with *L. monocytogenes* strains 6 h postinoculation. Bacteria were detected with anti-Lm (green), and bacteria actin tails and host actin were detected using phalloidin (red), while the cell nucleus was labeled with DAPI (blue). The scale bar is 10 µm. The high-magnification images displayed at the bottom of each image show F-actin (red), bacteria (green), and nuclei (blue).

Listeriolysin O plays an essential role in the escape of *L. monocytogenes* from both the primary phagosome and the secondary double-membrane-bound vesicle formed during cell-to-cell spreading (22). The data above did not directly address whether the LLO_N487V479_ mutation affects bacterial cell-to-cell spreading. Therefore, we examined the capability of these mutants to spread from cell to cell by measuring the diameter of plaques formed in L929 fibroblast monolayers after 3 days of infection in the presence of a low concentration of gentamicin. As indicated in (Figure 5A), no visible plaques were found from the cells infected with the avirulent Δ*hly* strain. Moreover, the compromised cell-to-cell spreading of the Δ*hly* strain was restored in the hemolytic strains CΔ*hly*, CΔ*hly*_N478A_, and CΔ*hly*_V479A_, and also in the non-hemolytic strain CΔ*hly*_N478AV479A_ (Figure 5A). Interestingly, these complemented strains, which exhibited the same spreading efficiency, showed a slight defect in terms of their plaque diameters, compared with the wild-type EGD-e strain, indicating that their compromised spreading ability was not fully complemented. Thus, the results suggest that *L. monocytogenes* expressing LLO_N487V479_ is not defective in cell-to-cell spreading. This was further confirmed in an actin-tail formation assay where the LLO mutant strains were able to associate with F-actin and formed long actin tails with an efficiency that was comparable to that of the wild-type EGD-e strain and the complemented strain CΔ*hly* in human epithelial Caco-2 cells (Figure 5B), while deletion of *hly* completely compromised the capability to spread from cell to cell, as expected (Figure 5B). Therefore, we conclude that LLO_N487V479_ is fully capable of mediating cell-to-cell spreading and escape from the double-membraned vesicle.

## Discussion

The pore-forming protein LLO is a primary determinant of *L. monocytogenes* pathogenesis that is important for bacterial vacuolar escape into the host cytosol. Tight restriction of its activity in the internalization vacuole appears to be important for infection. Undoubtedly, uncontrolled expression of LLO could lead to perforation of organelles and the host plasma membrane from the inside of the cell, causing cell death and destruction of the *L. monocytogenes* intracellular niche, thereby exposing the bacteria to the host immune system (8, 14). Various mechanisms responsible for tightly restricting the activity of LLO within the host cytoplasm have been investigated, such as its ubiquitylation and proteasomal degradation (22). The best studied strategy, however, is its sensitivity to pH and temperature. Replacement of LLO by pH-insensitive CDCs such as PFO from *Clostridium perfringens* or anthrolysin O from *Bacillus anthracis* allows phagosomal escape of *L. monocytogenes*, but leads to decreased infection (17, 23). In fact, the cytolytic activity of LLO does not correlate strictly with the capability of *Listeria* to escape from the vacuole. In this study, for the first time, we identified a novel mutant, LLO_N478AV479A_, which has barely detectable hemolytic activity, but which escaped, grew intracellularly, and spread cell-to-cell with the same efficiency as a strain secreting wild-type LLO. However, like bacteria lacking LLO, *L. monocytogenes* strains synthesizing LLO_N478AV479A_ were completely attenuated in virulence in mice. Thus, the results strongly suggest that these two residues at the C-terminus of LLO are key sites that are required for the hemolytic activity of LLO and essential for bacterial pathogenicity in mice, but they are not necessary for *L. monocytogenes* intracellular survival and cell-to-cell spreading.

The stability of LLO in the host cell cytosol is impacted by proteolytic degradation mechanisms that affect the ability of *L. monocytogenes* to cause infection. While sufficient levels of LLO are required to infect host cells and promote cell-to-cell spreading, abnormally high levels of LLO are linked to cellular toxicity and clearance of extracellular bacteria by innate immune mechanisms (24). Therefore, we firmly believe that the hemolytic activity and cytotoxicity of LLO must be limited to the lowest level to establish a successful infection within host cells. To achieve this, *L. monocytogenes* has evolved multiple mechanisms to finely modulate the cytolytic activity of LLO. *L. monocytogenes* expressing PFO was significantly impaired in its ability to grow intracellularly because the secreted PFO permeabilizes the host cell and allow gentamicin into the cell to kill intracellular bacteria. However, bacteria expressing mutated PFOs (T490I, G461D, and R468K), which have very low or undetectable hemolytic activity, were still capable of escaping from the phagocytic vacuole of macrophages, while their intracellular growth was slightly slower than that of a wild-type strain (17, 18). In contrast, previous studies have shown that deletion of the N-terminal PEST-like sequence does not affect LLO hemolytic activity, but it does cause a 1,000-fold decrease in intracellular growth and attenuate bacterial virulence, which is mainly ascribed to the fact that infection of host cells with LLOΔPEST-producing bacteria resulted in 90% of the maximal LDH release, compared with only 2% for wild-type bacteria. More importantly, bacteria synthesizing the PFO protein fused with the PEST motif can replicate intracellularly and are much less toxic to their host macrophages than bacteria producing the PFO protein without the PEST sequence, indicating that this motif restricts LLO activity to the host cell vacuole, thereby preserving the intracellular niche of *L. monocytogenes* (12). However, one puzzling observation showed that an LLO_L461T_ mutant, which displays high activity at neutral pH, still requires acidification of the vacuole to promote *Listeria* escape, suggesting the involvement of other factors in conjunction with LLO (25). In the present study, we demonstrated that the hemolytic activity of *L. monocytogenes* expressing a novel mutated LLO (N478AV479A) was completely impaired, but the bacteria were still able to grow intracellularly with a comparable efficiency to that of bacteria expressing wild-type LLO. Together with previously published data, these findings firmly support the view that *L. monocytogenes* has evolved multiple sophisticated mechanisms to minimize harm to host cells by regulating the activity of LLO. To establish a successful infection and achieve maximal virulence, this pathogen must maintain an equilibrium between producing LLO that is sufficiently cytolytic to escape from the vacuole, yet not overly toxic to infected host cells (25). LLO also has functions that are not linked to its pore-forming activity. Importantly, LLO activity is not unrestricted. Otherwise, host cells would not be able to survive infection by hundreds of bacteria, which is observed routinely. Instead, LLO activity is highly regulated by both *L. monocytogenes* and host cellular processes.

The LLO_N478AV479A_ mutant was capable of mediating vacuolar escape, growing intracellularly, and spreading cell-to-cell with an efficiency close to that of the wild-type strain. Moreover, the cytotoxic level of this mutant was very similar to that of the wild-type strain, indicating that this mutant form of LLO was toxic to the host cell at a normal level and, thus, was able to grow intracellularly both in epithelial cells and murine macrophages. Based on these results, we hypothesized that the virulence of the LLO_N478AV479A_ mutant would be comparable to that of its parent strain expressing wild-type LLO. Surprisingly, this mutant strain was completely non-virulent in mice, as was same the strain lacking LLO. A possible explanation for this result is that while the absence of the pore-forming ability of LLO may be an important protective mechanism, LLO may also have other properties that govern its function within a vacuole during host infection. Importantly, *L. monocytogenes* is able to infect both phagocytic and nonphagocytic cells, which results in potent innate and adaptive immune responses in an infected host that are required for the clearance of the organism (26). This ability to efficiently induce diverse, complex immune responses using multiple, simultaneous, and integrated mechanisms of action underlies the development of this bacterium as an antigen delivery vector to induce protective cellular immunity against cancer or viral infection (27). *L. monocytogenes* infection has been long known to also induce type I interferons (IFNs), IFN-α and IFN-β, which are usually associated with antiviral immune responses and essential for the immune system to clear many viral pathogens. In contrast to IFN-γ, type I IFNs are beneficial to *Listeria* infection (28–30). Induction of type I IFNs by *L. monocytogenes* requires escape of intracellular bacteria into the cytosol (31). Therefore, the highly attenuated *L. monocytogenes* mutants we identified were still capable of escaping into the cytosol, growing intracellularly, and inducing type I IFNs, potentially making these bacteria a promising tool for protecting against viral infection. However, *L. monocytogenes* has been shown to harness its ability to deliver foreign antigens efficiently to both the major histocompatibility class I and II presentation machinery and induce robust T-cell responses to *Listeria*-delivered antigens, thus making it a powerful vaccine vector for tumor immunotherapy. In a therapeutic setting, detoxified LLO with a completely impaired cytolytic activity acts as a potent adjuvant, enabling it to serve as a powerful antigen fusion partner to create antigen-adjuvant proteins (27). More recently, several studies have shown that tumor antigens genetically fused to detoxified LLO exhibit enhanced ubiquitin-proteasome-mediated processing and presentation by antigen-presenting cells for the activation of antigen-specific cytotoxic T lymphocytes, and stimulated the necessary proinflammatory responses for effective antitumor adaptive immune responses (32, 33). In the present study, we identified a novel, nontoxic LLO (N478AV479A) that still enables the bacteria to efficiently mediate vacuolar escape and survive intracellularly, while exhibiting attenuated virulence, which could provide a new adjuvant fusion partner with a cognate antigen for tumor immunotherapy. In conclusion, we have shown that residues N478 and V479 are required for the cytolytic activity of LLO and essential for *L. monocytogenes* pathogenicity in mice, but not for intracellular infection, which will provide new insights that increase our understanding of the current and future development of *Listeria*-based antigen delivery vectors to induce protective cellular immunity against tumors or infections.

## Materials and Methods

### Bacterial Strains, Plasmids, and Culture Conditions

*Listeria monocytogenes* EGD-e was used as the wild-type strain. *E. coli* DH5α was employed for cloning experiments and as the host strain for plasmids pET30a(+) (Merck, Darmstadt, Germany), pIMK2 and pKSV7. *E. coli* Rosetta (DE3) was used for prokaryotic protein expression. *Listeria* strains were cultured in brain–heart infusion (BHI) medium (Oxoid, Hampshire, England). *E. coli* strains were grown at 37°C in Luria-Bertani broth (LB) (Oxoid). Stock solutions of ampicillin (50 mg/mL), erythromycin (50 mg/mL), kanamycin (50 mg/mL), or chloramphenicol (50 mg/mL) were added to media where appropriate. All chemicals were obtained from Sangon Biotech (Shanghai, China), Merck or Sigma-Aldrich (St. Louis, MO, USA) and were of the highest purity available. All primers used in this study are listed in Table S1 in Supplementary Material.

### *L. monocytogenes* Gene In-Frame Deletion

The temperature-sensitive pKSV7 shuttle vector was used for creating mutations from *L. monocytogenes* strain EGD-e background. Genomic DNA was extracted as described previously (34, 35). A homologous recombination strategy with SOE-PCR procedure was used for in-frame deletion to construct *hly* deletion mutant (36). Specifically, the DNA fragments containing homologous arms upstream and downstream of *hly* were obtained by PCR amplification of EGD-e DNA templates using the SOE primer pairs *hly*-a/*hly*-b and *hly*-c/*hly*-d (Table S1 in Supplementary Material). The obtained fragment was then cloned into the vector pKSV7 and electroporated into the competent EGD-e cells. Transformants were grown at a non-permissive temperature (41°C) in BHI medium containing chloramphenicol to promote chromosomal integration and the recombinants were passaged successively in BHI without antibiotics at a permissive temperature (30°C) to enable plasmid excision and curing (37). The recombinants were identified as chloramphenicol-sensitive colonies, and the mutagenesis was further confirmed by PCR and DNA sequencing.

### Complementation of Gene Deletion Mutant

To complement the *L. monocytogenes* Δ*hly* strain, we constructed a complemented strain by using the integrative plasmid pIMK2. The complete open reading frame (ORF) of *hly* along with its native promoter region was amplified using the primer pairs CΔ*hly*-e/CΔ*hly*-f (Table S1 in Supplementary Material) and cloned into pIMK2 following restriction to cut off the *P*_help_ region with enzymes. The resulting plasmid was then electroporated into *L. monocytogenes* Δ*hly* strain. Regenerated cells were plated on BHI agar containing kanamycin (50 µg/mL). The complemented strain was designated as CΔ*hly*.

### Overexpression and Purification of His-Tagged LLO Proteins from *E. coli*

Recombinant proteins used in this study were expressed as fusion proteins to the N-terminal His-tag using pET30a(+) as the expression vector (38). *E. coli* Rosetta (DE3) was used as the expression host. The full-length ORF of the interest gene from EGD-e genome was amplified with the primer pair (Table S1 in Supplementary Material) and then inserted into the pET30a(+) vector, and finally transformed into Rosetta competent cells. *E. coli* cells harboring the recombinant plasmids were grown in 500 mL LB medium supplemented with 50 µg/mL kanamycin at 37°C until the cultures reached 0.8–1.0 at OD_600 nm_. Isopropyl β-D-1-thiogalactopyranoside (IPTG) was then added to a final concentration of 0.4 mM to induce expression of the recombinant proteins for additional 4 h under the optimized conditions. The His-tagged fusion proteins were purified using the nickel-chelated affinity column chromatography.

### Preparation of Polyclonal Antibodies against the Recombinant Proteins

Purified recombinant protein was used to raise polyclonal antibodies in New Zealand white rabbits according to a standard protocol (39). Briefly, rabbits were initially immunized *via* subcutaneous injection of 500 µg protein with an equal volume of Freund’s complete adjuvant (Sigma). After 2 weeks, rabbits were boosted by subcutaneous injection of 250 µg protein each in incomplete Freund’s adjuvant (Sigma) three times at 10-day intervals. Rabbits were bled ~10 days after the last injection.

### Site-Directed Mutagenesis

Single site-directed mutants (N478A and V479A) and the double site-directed mutant (N478AV479A) of LLO were generated using the original vector template, pET30a-LLO or pIMK2-LLO, and the QuikChange Site-Directed Mutagenesis kit (Agilent Technologies, Palo Alto, CA, USA) with the primer pairs described in Table S1 in Supplementary Material. Template DNA was removed *via* digestion with DpnI (Toyobo Co., Osaka, Japan) for 2 h at 37°C. All mutant constructs were sequenced to ensure that only the desired single mutations had been incorporated correctly. The mutant constructs based on the plasmid pET30a-LLO were transformed into *E. coli* Rosetta competent cells and the corresponding mutant proteins were designated as LLO_N478A_, LLO_V479A_, and LLO_N478AV479A_ accordingly, and expressed, purified as described above. For mutant complemented strains, the mutant constructs based on the plasmid pIMK2-LLO were electroporated into *L. monocytogenes* Δ*hly* competent cells, and the resultant strains were designated as CΔ*hly*_N478A_, CΔ*hly*_V479A_, and CΔ*hly*_N478AV479A_, respectively.

### Growth Analysis of *L. monocytogenes* in BHI Broth

*Listeria monocytogenes* wild-type strain EGD-e, mutant strain Δ*hly*, complemented strains CΔ*hly*, CΔ*hly*_N478A_, CΔ*hly*_V479A_, and CΔ*hly*_N478AV479A_ were grown overnight at 37°C in BHI broth with shaking. Cultures were collected by centrifugation at 5,000 × *g* at 4°C, washed once in phosphate-buffered saline (PBS) (10 mM, pH 7.4) and initial OD_600 nm_ adjusted to 1.0. Bacteria were diluted (1:100) in fresh BHI broth, and incubated at 37°C for 12 h. Kinetic growth was measured (OD_600 nm_) at 1-h interval.

### LLO-Mediated Hemolytic Activity Measurement

Measurement of LLO-associated hemolytic activity was done as described previously (40). *L. monocytogenes* wild-type and mutant strains were grown for 16 h with shaking in BHI broth at 37°C. All cultures were adjusted to OD_600 nm_ of 1.0 before supernatants were collected. Hemolytic activity was measured based on lysis of sheep red blood cells (SRBCs) of secreted LLO from culture supernatants. Specifically, culture supernatant (50 µL) was diluted in hemolysis buffer (10 mM PBS, pH 5.5 or 7.4, 150 mM NaCl, 1 mM DTT) in final volumes of 50 µL and equilibrated to 37°C for 10 min. Next, 50 µL PBS-washed intact SRBCs (5%) were added to each sample and incubated at 37°C for 30 min. Samples were centrifuged and supernatants analyzed for hemoglobin absorption at 550 nm. For hemolysis determination of recombinant proteins, purified LLO or its mutant protein (LLO_N478A_, LLO_V479A_, and LLO_N478AV479A_) expressed in *E. coli* was serially diluted in hemolysis buffer, then mixed with an equal volume of 5% SRBC and the hemolytic activity determined as described above. The values corresponding to the reciprocal of the dilution of culture supernatant required to lyse 50% of HRBCs were used to compare the hemolytic activities in the different supernatants. Erythrocytes incubated with 1% Triton X-100 or PBS served to determine the maximum (100%) and minimum (0%) hemolytic activity, respectively.

### Cell Fractionation and Protein Localization of LLO

Western blotting was employed to analyze the changes in expression of LLO. Bacterial overnight cultures of *L. monocytogenes* were diluted into 200 mL fresh BHI broth, and bacteria were grown to the stationary phase. For secreted proteins isolation: the fractionation procedure was described by Lenz and Portnoy (41), with minor modifications. Briefly, the bacteria cells were pelleted by centrifugation at 13,000 *g* for 20 min at 4°C, and the resulting culture supernatant collected and then filtered through a 0.22 µm polyethersulfone membrane filter (Thermo Fisher Scientific, Lafayette, LA, USA). Trichloroacetic acid (TCA) was added to the supernatant to reach a final concentration of 10% TCA. Proteins were TCA-precipitated on ice overnight and washed with ice-cold acetone. The washed precipitates of supernatant proteins were re-suspended in SDS-PAGE sample buffer (5% SDS, 10% glycerol, and 50 mM Tris–HCl, pH 6.8). Samples were boiled for 6 min and stored at −20°C before electrophoresis. For total cell proteins isolation: the previous method was applied (42). Specifically, the bacterial pellets were re-suspended in 1 mL of extraction solution (2% Triton X-100, 1% SDS, 100 mM NaCl, 10 mM Tris–HCl, 1 mM EDTA, pH 8.0). One gram of glass beads (G8772, Sigma-Aldrich) was added and samples lysed by using the homogenizer Precelly 24 (Bertin, Provence, France) at 6,000 rpm for 30 s with intermittent cooling for 30 s (3 cycles in total) and then centrifuged at 12,000 rpm for 15 min. Supernatant was retained as the whole cell extract. The method for total cell proteins isolation was used as described above. Protein samples were separated through a 12% SDS-PAGE and immunoblotted with α-LLO or α-GAPDH antisera. GAPDH was used as an internal control.

### Virulence in the Mouse Model

The *L. monocytogenes* wild-type strain EGD-e, mutant strain Δ*hly*, and complemented strains CΔ*hly*, CΔ*hly*_N478A_, CΔ*hly*_V479A_, and CΔ*hly*_N478AV479A_, were tested for recovery in liver and spleen sections of ICR mice (female, 18–22 g, purchased from Zhejiang Academy of Medical Sciences, Hangzhou, China) as previously described (39). The mice (8 per group) were injected intraperitoneally with ~10^6^ CFU of each strain. At 24 and 48 h postinfection, mice were sacrificed, and livers and spleens removed and individually homogenized in 10 mM PBS (pH 7.4). Surviving *Listeria* cells were enumerated by plating serial dilutions of homogenates on BHI agar plates. Results were expressed as means ± SD of recovery bacterial number (Log_10_ CFU) per organ for each group. For animal survival experiments, mice injected intraperitoneally with 1 × 10^7^ CFU listeria were monitored for up to 7 days after infection. Survival curves were calculated by using the Kaplan–Meier method and differences in survival were determined by using the Log-rank test.

### Proliferation in RAW264.7 Macrophages

Bacteria survival or proliferation murine macrophages RAW264.7 was conducted as previously described (43). Stationary *L. monocytogenes* wild-type strain EGD-e, mutant Δ*hly*, complemented strains CΔ*hly*, CΔ*hly*_N478A_, CΔ*hly*_V479A_, and CΔ*hly*_N478AV479A_ cells at 37°C in BHI were washed and re-suspended in 10 mM PBS (pH 7.4). Monolayers of RAW264.7 cells, cultured in Dulbecco’s modified Eagle medium (DMEM, Thermo Fisher Scientific) containing 10% fetal bovine serum (FBS, GE Healthcare Hyclone, Logan, UT, USA) were infected with the above strains for 60 min with multiplicity of infection (MOI) at 10:1. For adhesion, cells were lysed after being washed three times with PBS. For estimation of invasion, cells were washed with PBS after 1 h infection and incubated for an additional hour in DMEM containing gentamicin at 50 µg/mL for 30 min to kill extracellular bacteria. Infected cells were lysed by adding 1 mL of ice-cold sterile distilled water. The lysates were 10-fold diluted for enumeration of viable bacteria on BHI agar plates that were considered as the 0 h numbers invading into the cells. For intracellular proliferation, cells were subjected to further incubation for 6, 12, or 18 h in 5% CO_2_ at 37°C. Viable bacteria were enumerated by serial dilution and colony counting on BHI agar plates.

### Intracellular Growth in J774 Macrophages

Intracellular growth was performed as described previously for monolayers of J774 macrophages (44). Specifically, overnight cultures of *L. monocytogenes* strains were washed three times and re-suspended in PBS, and then J774 cells infected with bacteria at an MOI of 0.05. Following 30 min incubation, cells were washed, and extracellular bacteria were killed by adding complete medium containing 50 µg/mL of gentamicin for an additional 30 min incubation. At 2, 6, or 12 h postinfection, cells were washed with PBS and finally lysed in ice-cold sterile distilled water. The number of viable intracellular *L. monocytogenes* cells was calculated by serial dilution and colony counting on BHI agar plates as mentioned above.

### Plaque Assay

The plaque assay was carried out by conventional methods (45, 46). Briefly, mouse L929 fibroblast cell monolayers were maintained in high-glucose DMEM medium (Thermo Fisher Scientific) plus FBS (Hyclone) and 2 mM l-glutamine. Cells were plated at 1 × 10^6^ cells per well in a six-well dish and infected at an MOI of 1:50 with *L. monocytogenes* at 37°C with 5% CO_2_ for 1 h. Extracellular bacteria were killed with 100 µg/mL gentamicin, and the cells washed three times with 10 mM PBS (pH 7.4) and then overlaid with 3 mL of medium plus 0.7% agarose and 10 µg/mL gentamicin. Following a 72-h incubation at 37°C, cells were fixed with paraformaldehyde (4% in PBS for 20 min) and stained with crystal violet. The diameter of plaques was measured by Adobe Photoshop software for each strain. The plaque size of wild-type strain EGD-e was set as 100% and data are shown as means ± SDs.

### Phagosomal Escape Assay in Caco-2 Cells

Phagosomal escape assay was conducted according to the previous work (39). Specifically, human intestinal epithelial Caco-2 cells were infected at MOI of 10:1 at 37°C with 5% CO_2_ for 1 h. Extracellular bacteria were then killed with 50 µg/mL gentamicin for 1 h and incubated for an additional 6 h. Cells were washed gently with PBS (10 mM, pH 7.4), fixed with 4% paraformaldehyde and then permeabilized with 0.5% Triton X-100. The bacterial cells were stained with polyclonal antibodies to *L. monocytogenes* for 1 h at 37°C, washed twice with PBS, and probed with Alexa Fluor 488-conjugated donkey anti-rabbit antibody (Santa Cruz) for 1 h at 37°C. F-actin was then stained with phalloidin-Alexa Fluor 568 (Thermo Fisher Scientific). DAPI (4′,6-diamidino-2-phenylindole) (Thermo Fisher Scientific) was used to stain the nuclei. Actin tails recruited by the bacteria were visualized under a ZEISS LSM510 confocal microscope (Zeiss Germany, Oberkochen, Germany).

### Cytotoxicity Detection

Cytotoxicity was detected based on LDH release from J774 macrophages following bacterial infection by using the CytoTox 96 non-radioactive cytotoxicity assay kit according to the manufacturer’s instructions (Promega, WI, USA), as previously described by Decatur and Portnoy (12). Overnight cultured *L. monocytogenes* was deposited onto J774 cells at a MOI of 10 at 37°C with 5% CO_2_ for 30 min, after which the culture medium with or without 50 µg/mL gentamicin was added. To determine maximum LDH release, 100 µL of lysis buffer was added to triplicate infected wells 45 min prior to LDH measurement. At the indicated infection times (2, 4, and 6 h), cells were centrifuged at 250 × *g* for 5 min, and the supernatant was removed and used for the LDH assay. The supernatant was incubated for 30 min with 50 µL substrate mix prior to the addition of 50 µL stop solution. Absorption at 490 nm was then measured using the Micro-plate reader Synergy H1 (BioTek Solutions, Inc., Santa Barbara, CA, USA). The experimental design included three wells containing only DMEM to account for background absorption as well as three wells containing uninfected J774 cells to measure spontaneous LDH release. After background correction, the percent cytotoxicity was calculated as follows: cytotoxicity% = [(experimental LDH release − spontaneous LDH release)/(maximum LDH release − spontaneous LDH release)] × 100.

### Statistical Analysis

All experiments were repeated at least three times. Data were analyzed using the two-tailed homoscedastic Student’s *t*-test. Differences with *P-*values <0.05 were considered as statistically significant.

## Ethics Statement

All animal care and use protocols were performed in accordance with the Regulations for the Administration of Affairs Concerning Experimental Animals approved by the State Council of People’s Republic of China. The protocol was approved by the Institutional Animal Care and Use Committee of Zhejiang A&F University (Permit Number: ZJAFU/IACUC_2011-10-25-02). All the *Listeria monocytogenes*-involved experiments in our study were conducted at Biosafety Level 2 laboratory.

## Author Contributions

CC, WF, and HS conceived the study. CC, LJ, TM, JS, HW, XH, YH, HY, CF, and FL carried out experiments. CC, YY, ZC, and HH analyzed data. CC, NF, and HS drafted the manuscript and all authors contributed to this study prepared the final version of the manuscript. All authors read and approved the final manuscript.

## Conflict of Interest Statement

The authors declare that the research was conducted in the absence of any commercial or financial relationships that could be construed as a potential conflict of interest.
