# Supplementary material for: Carboxyl-Terminal Residues N478 and V479 Required for the Cytolytic Activity of Listeriolysin O Play a Critical Role in Listeria monocytogenes Pathogenicity
Source: Front Immunol. 2017 Nov 1;8:1439. doi: 10.3389/fimmu.2017.01439 (PMC5671954; doi:10.3389/fimmu.2017.01439)
Supplement: Supplementary file 1 [file Table_1.PDF]

Table S1. PCR Primers used in this study. Nucleotides introduced to create restriction enzyme sites are underlined. All primers were synthesized by Sangon Biotech, Inc., Shanghai, China.

**Primer name**	**Primer sequence (5'-3')**	**Product (bp)**	**Description**
hly-a	ACGCGTCGACTTCCTGCTTCTAGTTGTTGGTACAATGA	519	Used for construction of *hly* null mutant strain
hly-b	TATTACTTTTACAAGGGTTTCACTCTCCTTCTACATTTTTTAAC		
hly-c	AGAGTGAAACCCTTGTAAAAGTAATAAAAAATTAAGAATAAAACCGC	518	
hly-d	CGCGGATCCGCTTATCTTTTGTCATATCAAGAATTTTAAAATTT		
hly-a-front	TGAGTAATCGTTTCTAATACACCTGAAAGTG		
CΔ*hly*-e	CGCGAGCTCAATGGCCCCCTCCTTTGATTAGTATA	1802	Used for complementation of the *hly* deletion mutant under P_{hly}
CΔ*hly*-f	GCACTGCAGTTATTCGATTGGATTATCTACTTTATTACTATATTTCG		
LLO-exp-fwd	GGAATTCCATATGAAGGATGCATCTGCATTCAATAAAG	1520	Used for recombinant LLO expression in *E. coli*
LLO-exp-rev	CCGCTCGAGTTCGATTGGATTATCTACTTTATTACTATATTTCG		
LLO_N478A-fwd	AACGCGAGAAATATTGCTGTTTACGCTAAAGAATGCACT	6756	Used for site-directed mutagenesis of LLO
LLO_N478A-rev	AGTGCATTCTTTAGCGTAAACAGCAATATTTCTCGCGTT		
LLO_V479A-fwd	AACGCGAGAAATATTAATGCTTACGCTAAAGAATGCACT	6756	
LLO_V479A-rev	AGTGCATTCTTTAGCGTAAGCATTAATATTTCTCGCGTT		
LLO_N478AV479A-fwd	AACGCGAGAAATATTGCTGCTTACGCTAAAGAATGCACT	6756	
LLO_N478AV479A-rev	AGTGCATTCTTTAGCGTAAGCAGCAATATTTCTCGCGTT		
