# Supplementary figures and images for: Carboxyl-Terminal Residues N478 and V479 Required for the Cytolytic Activity of Listeriolysin O Play a Critical Role in Listeria monocytogenes Pathogenicity
Source: Front Immunol. 2017 Nov 1;8:1439. doi: 10.3389/fimmu.2017.01439 (PMC5671954; doi:10.3389/fimmu.2017.01439)

**Figure S1**

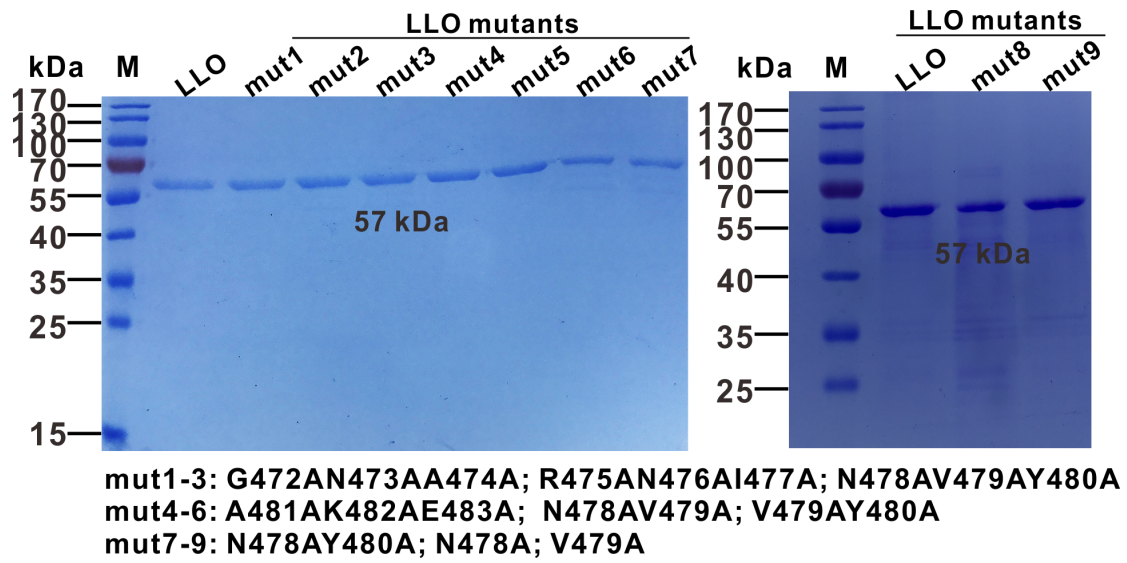

**Figure S1.** The original images of SDS-PAGE gels in Fig. 1B.

Supplement: Supplementary file 2 [file Image_1.PDF]

**Figure S2**

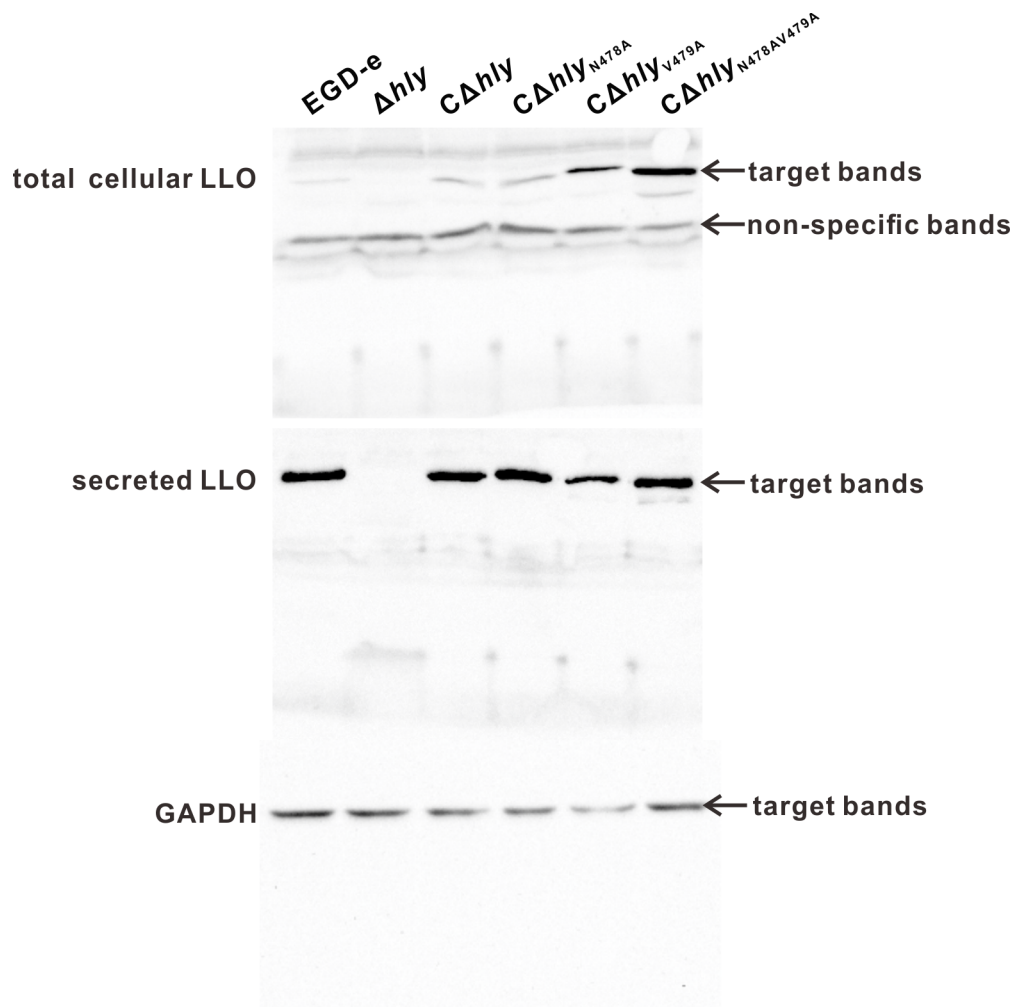

**Figure S2. The original images of Western Blots in Fig. 2A.**

Supplement: Supplementary file 3 [file Image_2.PDF]
